# Supplementary material for: Signal transduction in light-oxygen-voltage receptors lacking the active-site glutamine
Source: Nat Commun. 2022 May 12;13:2618. doi: 10.1038/s41467-022-30252-4 (PMC9098866; doi:10.1038/s41467-022-30252-4)
Supplement: Supplementary file 1 — Supplementary Information [file 41467_2022_30252_MOESM1_ESM.pdf]

Supplementary Information

Signal Transduction in Light-Oxygen-Voltage Receptors Lacking the Active-Site Glutamine

Julia Dietler[1,#], Renate Gelfert[1,#], Jennifer Kaiser[1,#], Veniamin Borin[2], Christian Renzl[3], Sebastian Pilsl[3], Américo Tavares Ranzani[1], Andrés García de Fuentes[1], Tobias Gleichmann[4], Ralph P. Diensthuber[4], Michael Weyand[1], Günter Mayer[3,5], Igor Schapiro[2], and Andreas Möglich[1,4,6,7,†,*]

[1] Department of Biochemistry, University of Bayreuth, 95447 Bayreuth, Germany
[2] Institute of Chemistry, The Hebrew University of Jerusalem, Jerusalem, Israel
[3] Life and Medical Sciences (LIMES), University of Bonn,53121 Bonn, Germany
[4] Biophysical Chemistry, Humboldt-University Berlin, 10115 Berlin, Germany
[5] Center of Aptamer Research & Development, University of Bonn, 53121 Bonn, Germany
[6] Bayreuth Center for Biochemistry & Molecular Biology, Universität Bayreuth, 95447 Bayreuth, Germany
[7] North-Bavarian NMR Center, Universität Bayreuth, 95447 Bayreuth, Germany

[#] These authors contributed equally.

[†] ORCID identifiers: J.D. 0000-0002-0418-0796; R.G. 0000-0002-2677-5774; J.K. 0000-0003-3029-3505; V.B. 0000-0001-7832-1443; C.R. 0000-0003-2296-1825; S.P. 0000-0003-1063-1407; A.T.R. 0000-0002-6203-9467; A.G.F. 0000-0002-0072-4725; R.P.D. 0000-0002-4864-7001; M.W. 0000-0002-7499-1324; G.M. 0000-0003-3010-4049; I.S. 0000-0001-8536-6869; A.M. 0000-0002-7382-2772

[*] To whom correspondence should be addressed. Tel: +49-921-55-7835; Email: andreas.moeglich@uni-bayreuth.de

Supplementary Data

1. zipped MTZ file for calculation of F_{light}-F_{dark} difference electron density map of *As*LOV2 wild-type
2. zipped MTZ file for calculation of F_{light}-F_{dark} difference electron density map of *As*LOV2 Q513L
3. PDF file with multiple sequence alignment of LOV$^{\Delta Q}$ receptors lacking the conserved glutamine

Supplementary Figures

a

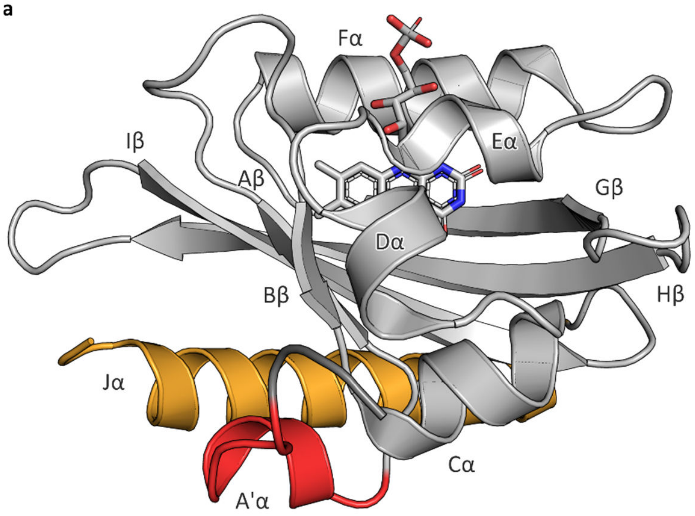

b Key residues

*As*LOV2	*Nm*PAL	YF1 (*Bs*YtvA)
N414	A248	G26
C450	C284	C62
N482	N316	N94
N492	N326	N104
Q513	Q347	Q123
D515	D349	D125

Suppl. Fig. 1 - Overview of tertiary structure and key residues in model light-oxygen-voltage (LOV) receptors. **a**, Three-dimensional structure of *As*LOV2 in its dark-adapted state (PDB 7pgx [http://doi.org/10.2210/pdb7pgx/pdb], this work). Secondary structure elements are labeled, and the terminal helices A'α and Jα are highlighted in red and orange, respectively. **b**, The table lists key residues in *As*LOV2[1] and the structurally equivalent residues in the *Nm*PAL[2] and YF1[3] receptors (see Fig. 1b). Note that the chimeric receptor YF1 comprises the *Bs*YtvA LOV domain[4].

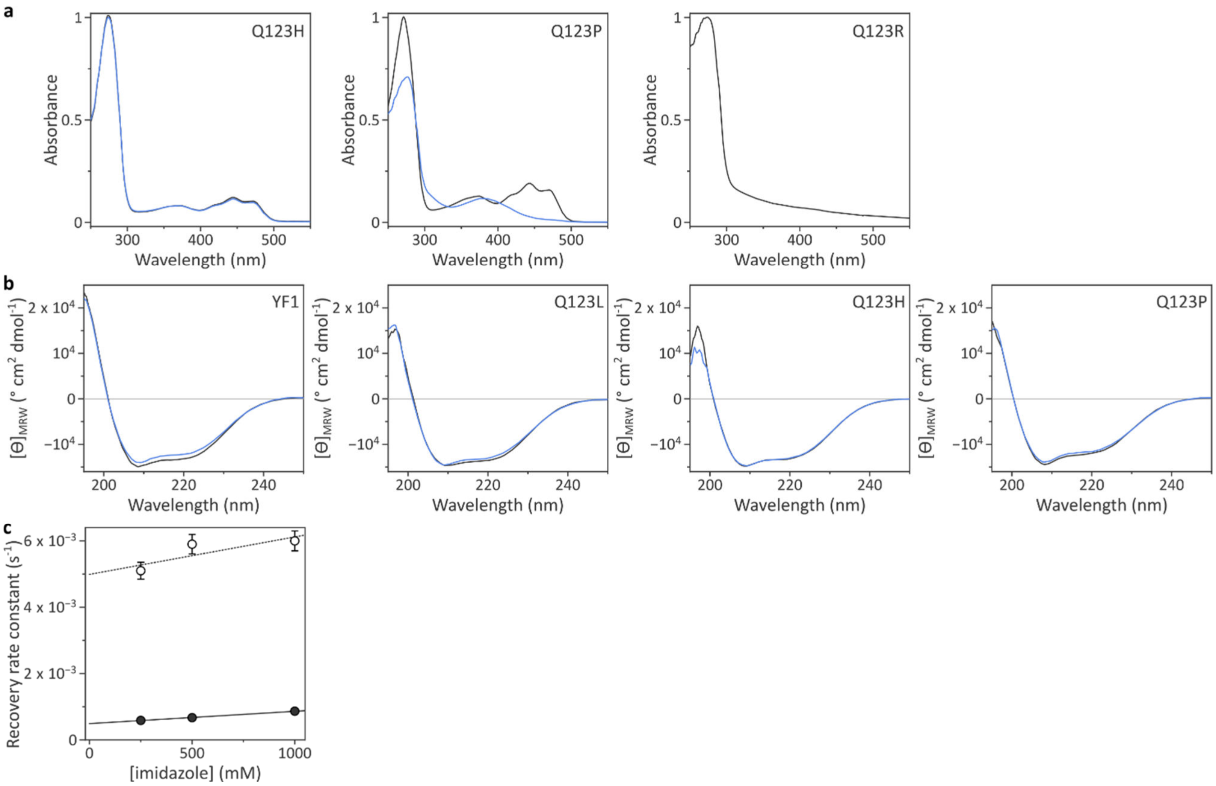

Suppl. Fig. 2 - Spectral analysis of YF1 variants. **a**, Absorbance spectra of the YF1 variants Q123H, Q123P, and Q123R in their dark-adapted states (black) and light-adapted states (blue). **b**, Far-UV circular dichroism spectra of YF1 and its variants Q123L, Q123H, and Q123P in their dark-adapted (black) and light-adapted states (blue). **c**, Rate constants for dark recovery of YF1 (open circles) and the Q123L variant (filled circles) determined at 37°C and varying imidazole concentrations[5]. Extrapolated to zero imidazole, recovery rate constants k_{-1} of (5.05 ± 0.05) × 10^{-3} s^{-1} and (4.91 ± 0.09) × 10^{-4} s^{-1} are obtained for YF1 and Q123L, respectively.

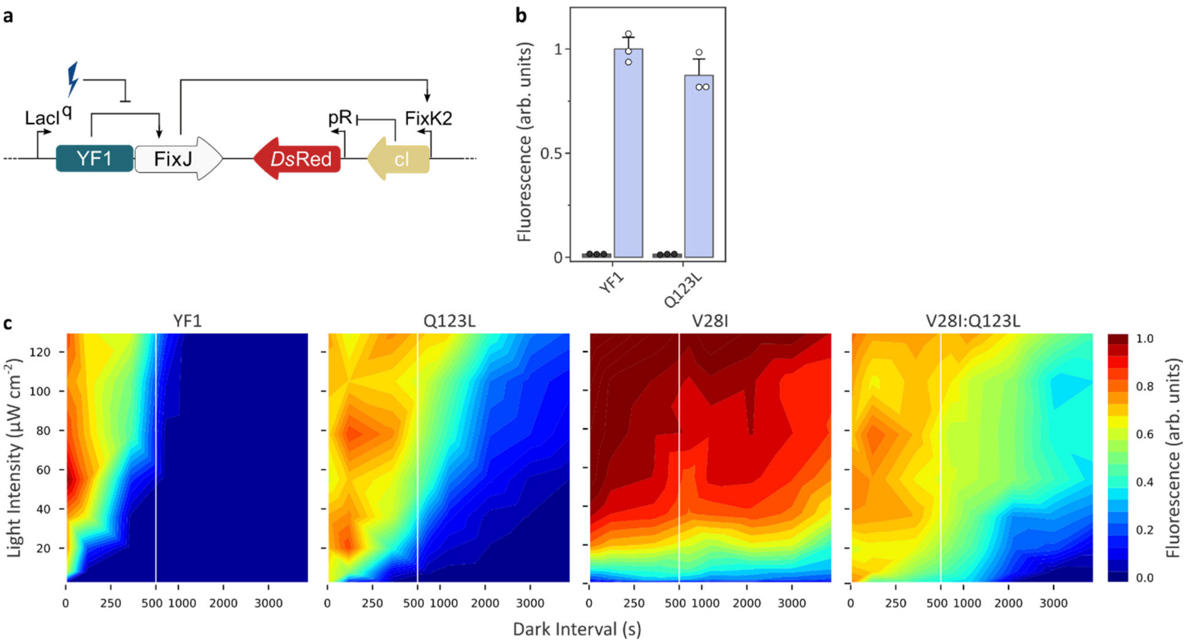

Suppl. Fig. 3 - Activity and light response of YF1 variants, assessed in the pDawn-*Ds*Red system[6]. **a**, The pDawn plasmid derives from pDusk (see Fig. 2a) and harbors an additional inversion cassette based on the lambda phage repressor cI. Expression of the *Ds*Red reporter is hence promoted by blue light rather than repressed. **b**, Normalized *Ds*Red fluorescence of *E. coli* cultures harboring pDawn plasmids encoding YF1 or the Q123L variant. Cells were cultivated in darkness (black dots and grey bars) or under constant blue light (white dots and blue bars). Data represent mean ± s.d. of three biologically independent replicates. **c**, *E. coli* cultures harboring pDawn plasmids encoding different YF1 variants were exposed to pulsatile blue light of varying intensity (ordinate)[7]. Half-minute periods of illumination alternated with dark intervals of differing duration (abscissa). Normalized fluorescence values represent mean ± s.d. of three biologically independent replicates. The experiments were repeated at least twice with similar outcome.

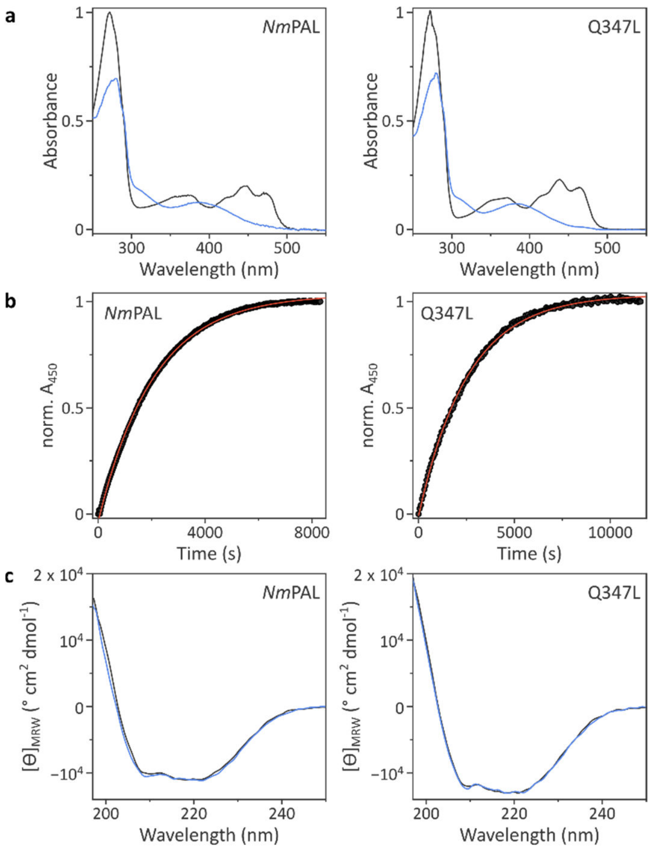

Suppl. Fig. 4 - Spectral analysis of *Nm*PAL variants. **a**, Absorbance spectra of *Nm*PAL wild-type and Q347L in their dark-adapted states (black) and light-adapted states (blue). **b**, Dark recovery of *Nm*PAL wild-type and Q347L after blue light ceases, monitored at a wavelength of 450 nm, respectively. Data were fitted to a single-exponential decay (red lines), yielding recovery rate constants k_{-1} of (4.86 ± 0.02) × 10^{-4} s^{-1} and (3.92 ± 0.02) × 10^{-4} s^{-1} for *Nm*PAL wild-type and Q347L, respectively. **c**, Far-UV circular dichroism spectra of *Nm*PAL wild-type and Q347L in their dark-adapted (black) and light-adapted states (blue).

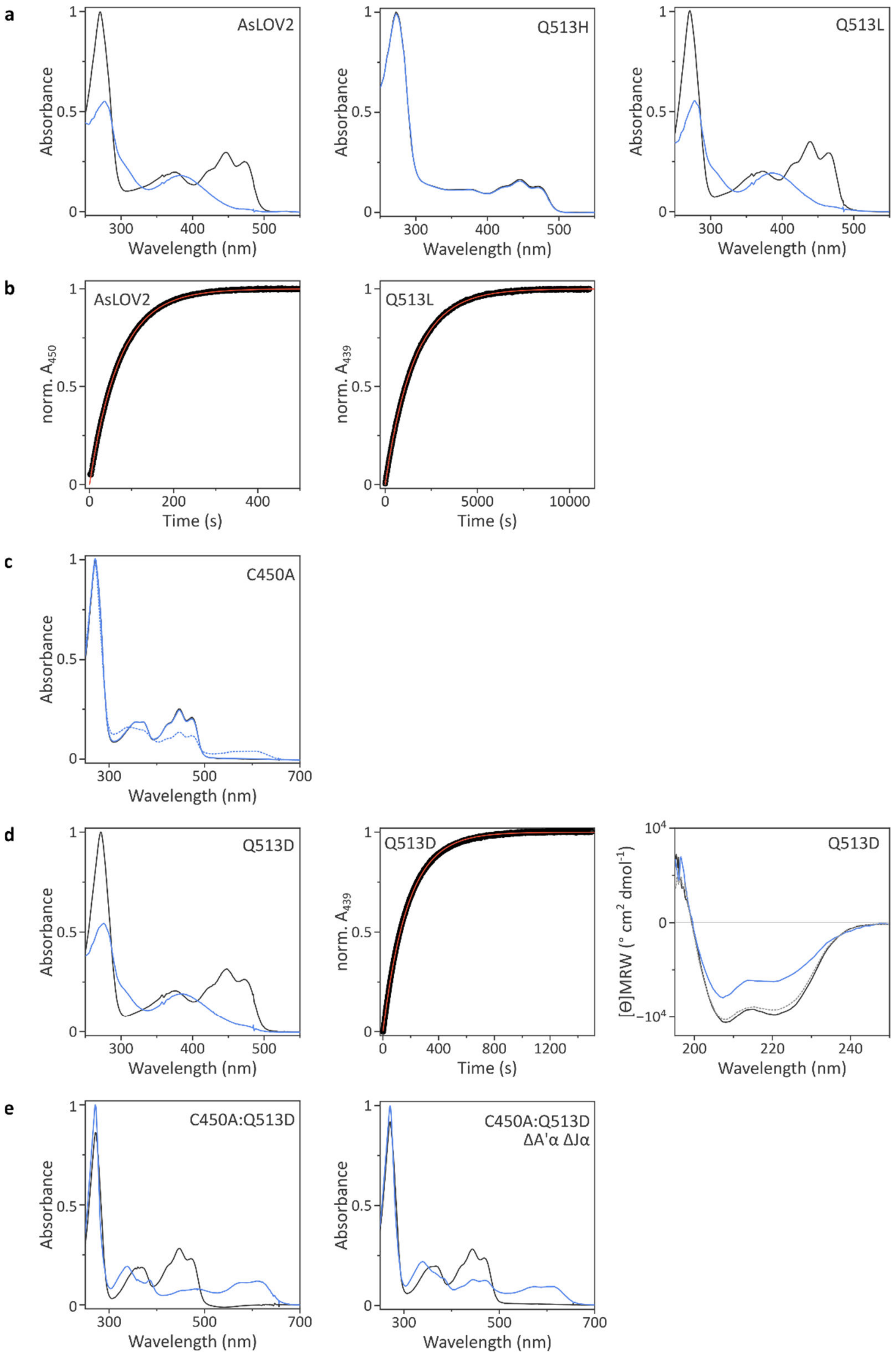

Suppl. Fig. 5 - Spectral analysis of *As*LOV2 variants. **a**, Absorbance spectra of *As*LOV2 wild-type, Q513H, and Q513L in their dark-adapted states (black) and light-adapted states (blue). **b**, Dark recovery of *As*LOV2 wild-type and Q513L after blue light ceases, monitored at wavelengths of 450 nm and 439 nm, respectively. Data were fitted to a single-exponential decay (red lines), yielding recovery rate constants k_{-1} of $(1.41 \pm 0.01) \times 10^{-2}$ s^{-1} and $(6.37 \pm 0.01) \times 10^{-4}$ s^{-1} for *As*LOV2 wild-type and Q513L, respectively. **c**, Absorbance spectra of *As*LOV2 C450A in the presence of 1 mM TCEP in its dark-adapted state and after illumination (470 nm, 20 mW cm^{-2}) for 30 s (solid blue line) and 5 min (dotted blue line). **d**, As in panels a and b, absorbance spectra and recovery kinetics for the *As*LOV2 Q513D variant; the recovery rate

constant k_{-1} amounted to (5.67 ± 0.01) × 10^{-3} s^{-1}. (right) Far-UV circular dichroism spectra of *As*LOV2 Q513D in its dark-adapted (black) and light-adapted states (blue). **e**, Absorbance spectra of *As*LOV2 C450A:Q513D (left) and C450A:Q513D ΔA'α ΔJα (right) in the dark-adapted state and after illumination (as in panel c) with no reductant added.

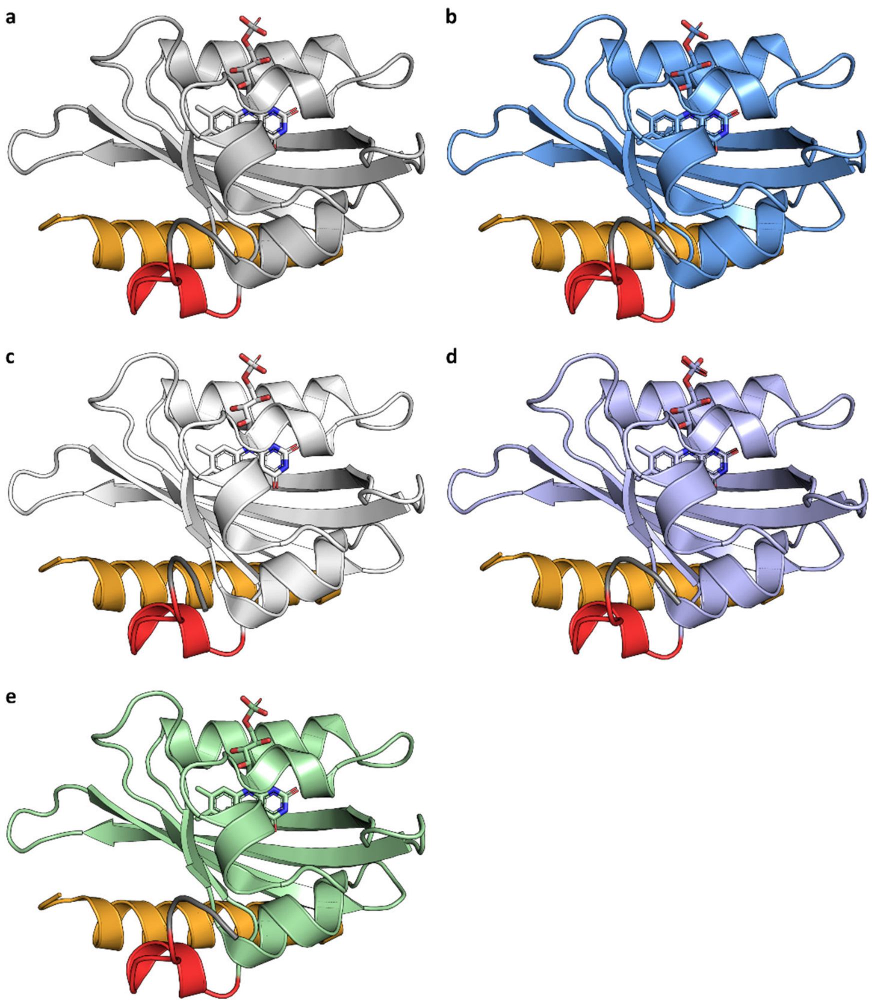

Suppl. Fig. 6 - Structural analyses of *As*LOV2 variants. **a**, Wild-type *As*LOV2 in its dark-adapted state as revealed by a 1.00 Å crystal structure. **b**, Wild-type *As*LOV2 in its light-adapted state as revealed by a 1.09 Å crystal structure. **c**, *As*LOV2 Q513L in its dark-adapted state as revealed by a 0.90 Å crystal structure. **d**, *As*LOV2 Q513L in its light-adapted state as revealed by a 0.98 Å crystal structure. **e**, The previously determined crystal structure of wild-type AsLOV2 (PDB 2v0u[8]) at 1.40 Å resolution. The flavin mononucleotide cofactors are shown in stick representation, and the A'α segment and the Jα helix are drawn in red and orange, respectively; N-terminal tags resulting from purification are shown in dark grey.

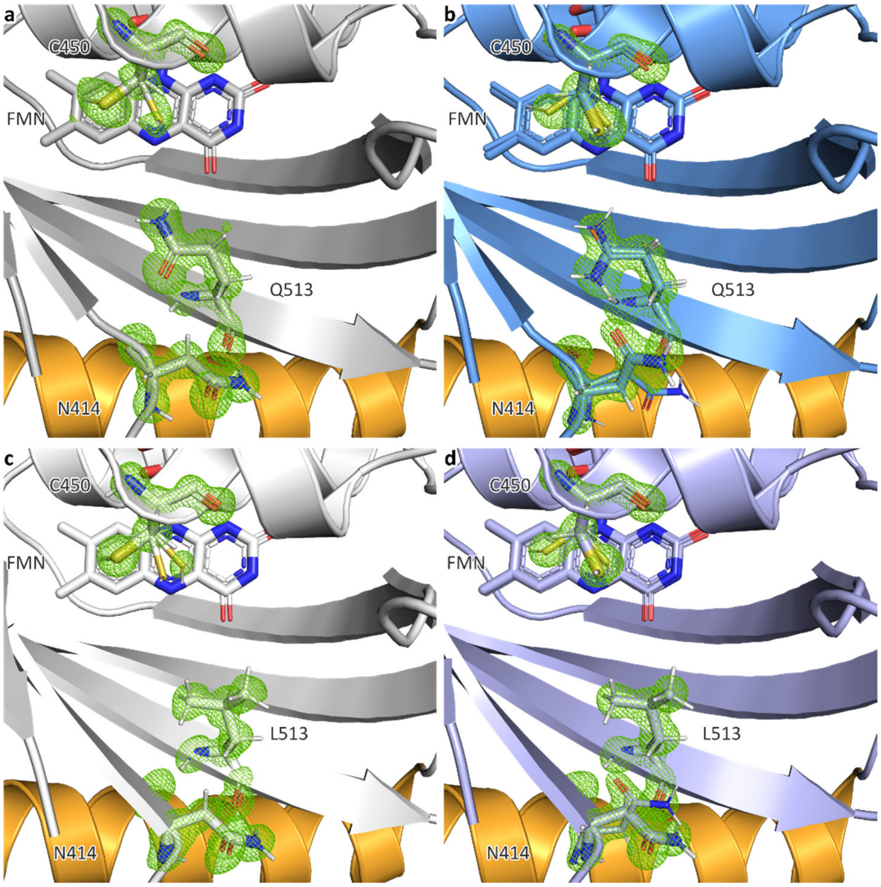

Suppl. Fig. 7 - F_o-F_c omit maps calculated upon setting the occupancy of residues N414, C450, and Q513 to zero. The green mesh denotes a contour level of +3.0 σ. **a**, Wild-type *As*LOV2 in its dark-adapted state. **b**, Wild-type *As*LOV2 in its light-adapted state. **c**, *As*LOV2 Q513L in its dark-adapted state. **d**, *As*LOV2 Q513L in its light-adapted state.

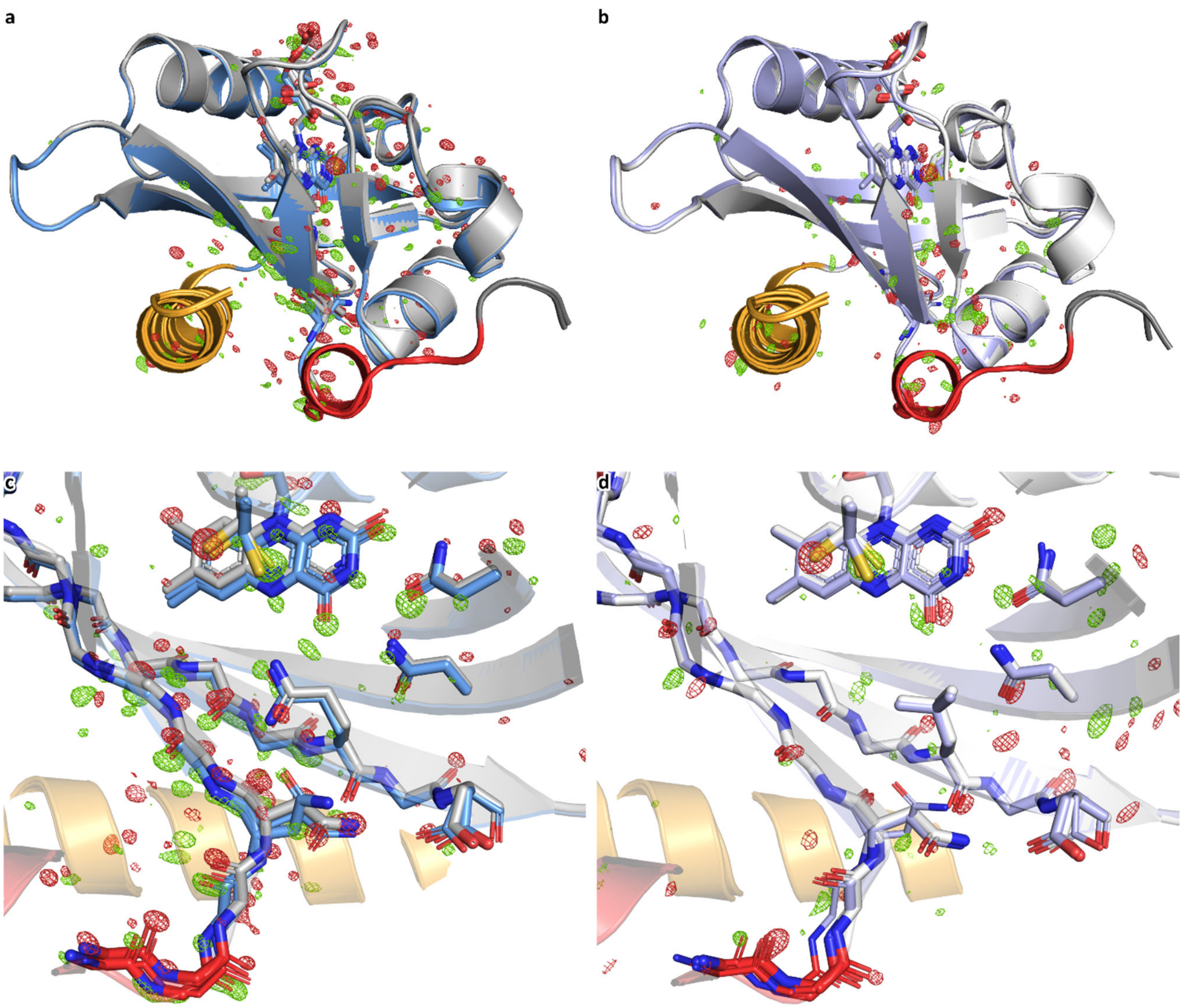

Suppl. Fig. 8 - F_{light}-F_{dark} difference electron density maps of *As*LOV2 wild-type and Q513L contoured at ±4.0 σ. The green mesh denotes regions with higher electron density in the light-adapted than the dark-adapted state. Vice versa, the red mesh marks areas with higher electron density in the dark-adapted state. The difference maps were calculated with phase information of the (better-resolved) dark-adapted data sets. **a**, The light-dark difference density in wild-type *As*LOV2 concentrates on the FMN chromophore, the strands Aβ and Hβ, and the helices A'α, Cα, Dα, Eα, and Jα. **b**, *As*LOV2 Q513L exhibits similar light-dark difference density maps, albeit at lower amplitude than in the wild-type protein. **c**, Close-up view of the chromophore-binding pocket in *As*LOV2 wild-type. **d**, Close-up view of the chromophore-binding pocket in *As*LOV2 Q513L.

a b

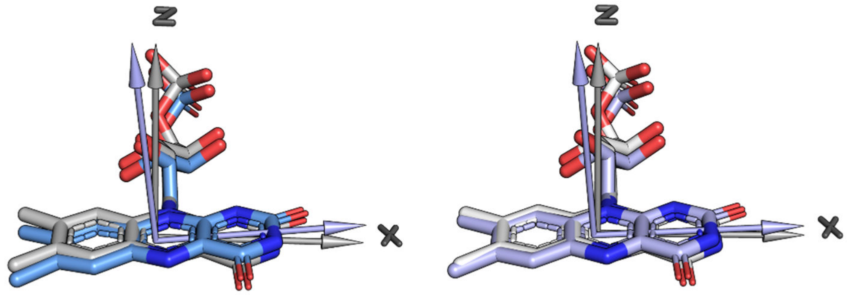

Suppl. Fig. 9 - Tilting of the flavin cofactor in the light-adapted state. **a**, Structures of the dark-adapted (grey) and light-adapted states (blue) of *As*LOV2 wild type were superposed. The flavin isoalloxazine plane tilts by around ~ 6.9° in the light-adapted state relative to the dark-adapted one. **b**, As in a but for *As*LOV2 Q513L and with a plane tilt of ~ 4.6°.

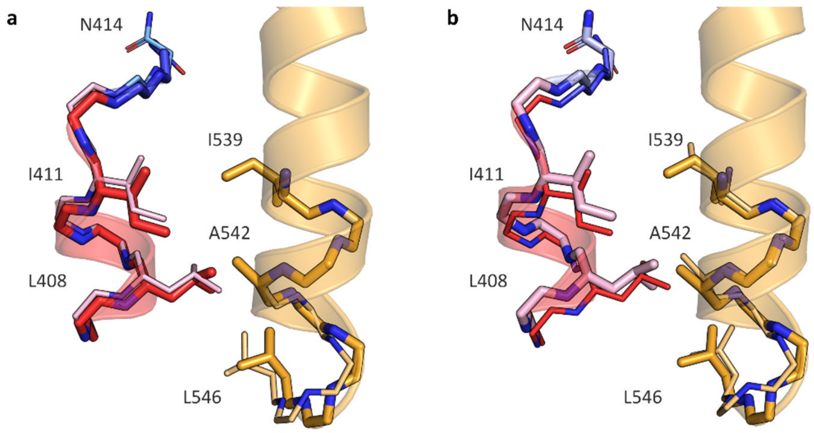

Suppl. Fig. 10 - Conformational transitions in *As*LOV2 wild type (panel a) and Q513L (panel b), as observed in the structures of the respective light-adapted states. Selected sidechains and the backbone of the A'α segment and the Jα helix are shown in stick representation. Minor conformations are drawn with narrower diameter. The A'α conformation corresponding to the dark-adapted state is shown in light pink.

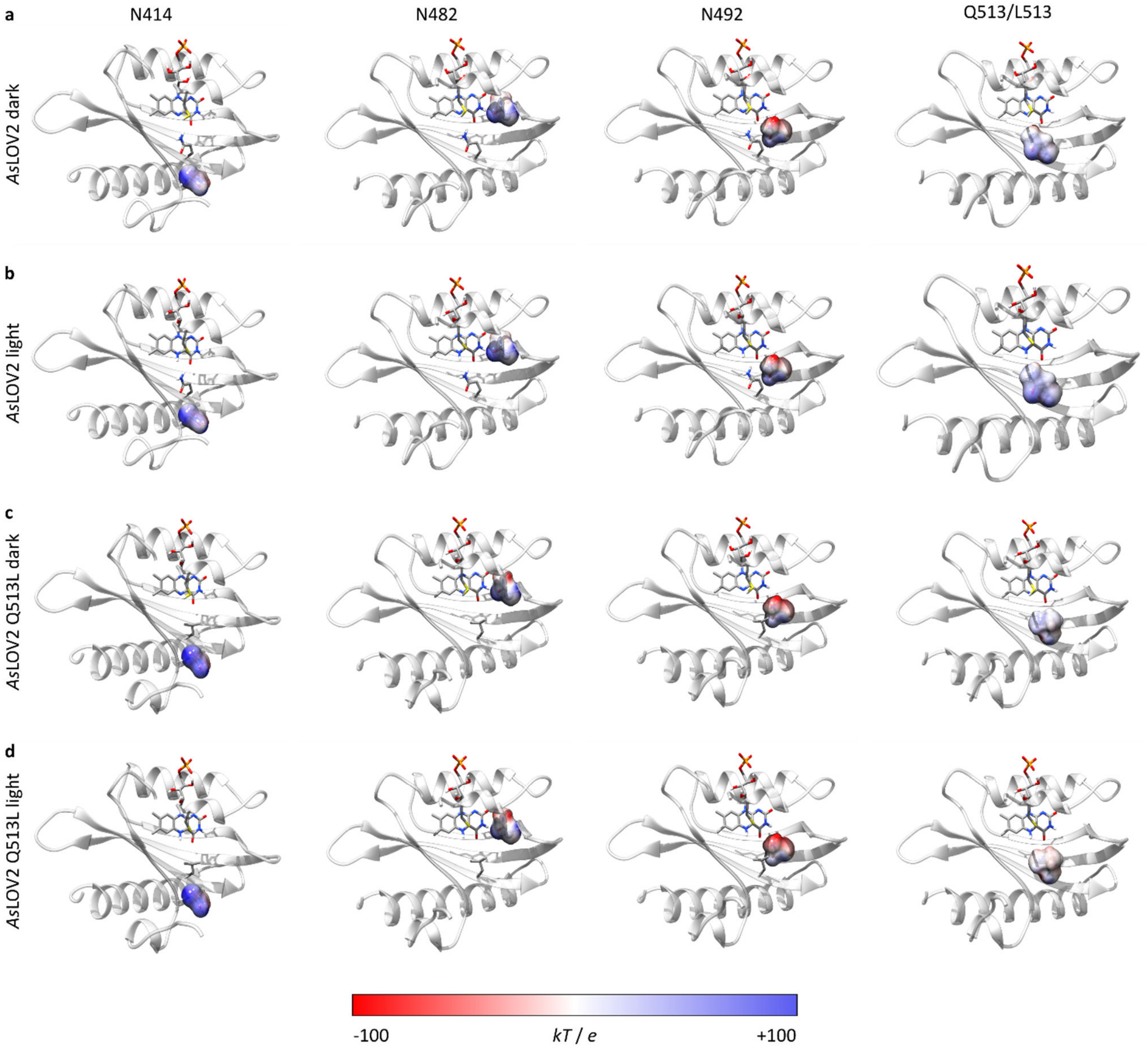

Suppl. Fig. 11 - Electrostatic potential acting on selected residues in *As*LOV2 wild-type and Q513L. **a**, Electrostatic potential acting on N414, N482, N492, and Q513 within wild-type *As*LOV2 in its dark-adapted state. **b**, As panel a but for light-adapted *As*LOV2 wild-type. **c**, As panel a but for residues N414, N482, N492, and L513 in dark-adapted *As*LOV2 Q513L. **d**, As panel c but for light-adapted *As*LOV2 Q513L.

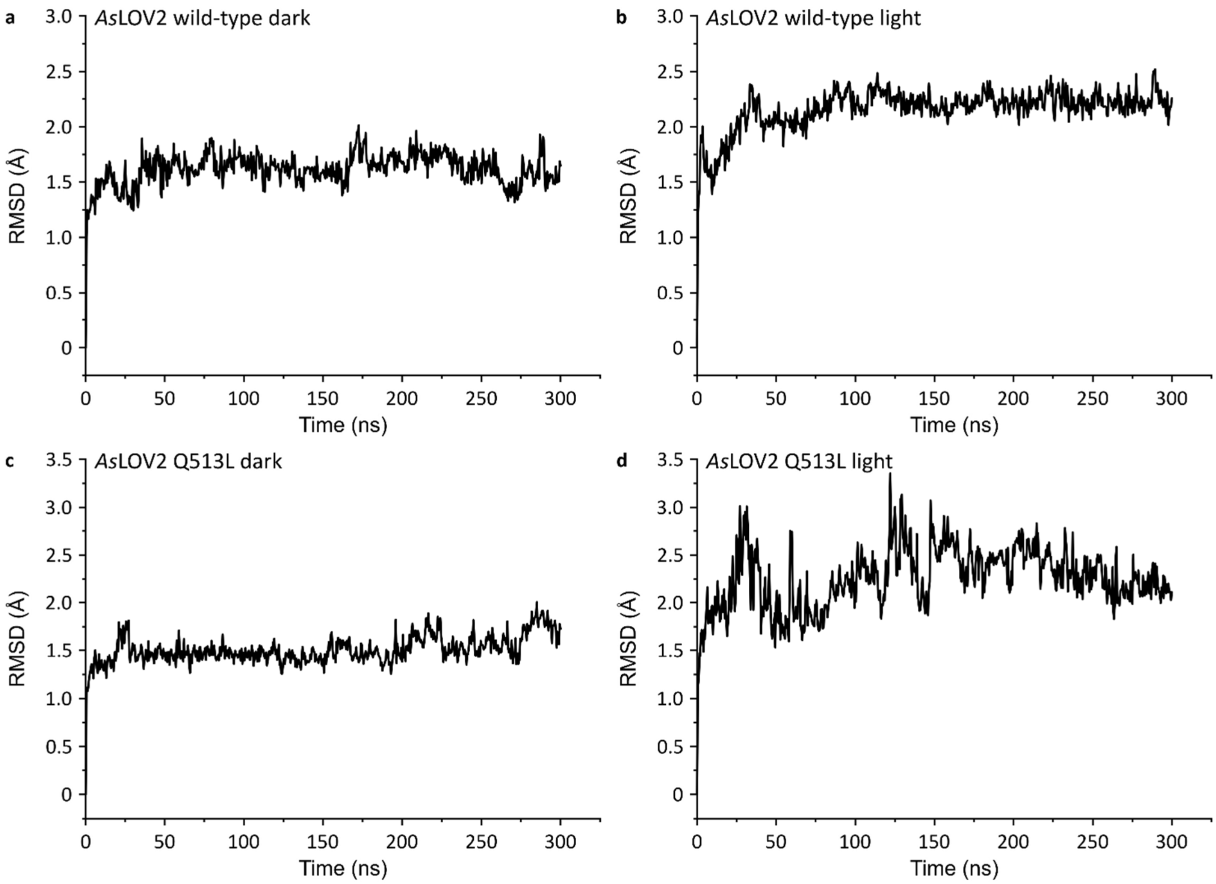

Suppl. Fig. 12 - RMSD variation along the molecular dynamics simulations of *As*LOV2 wild-type dark-adapted (panel **a**), *As*LOV2 wild-type light-adapted (panel **b**), *As*LOV2 Q513L dark-adapted (panel **c**), and *As*LOV2 Q513L light-adapted (panel **d**). RMSD values are relative to the starting structure and were calculated for all heavy atoms.

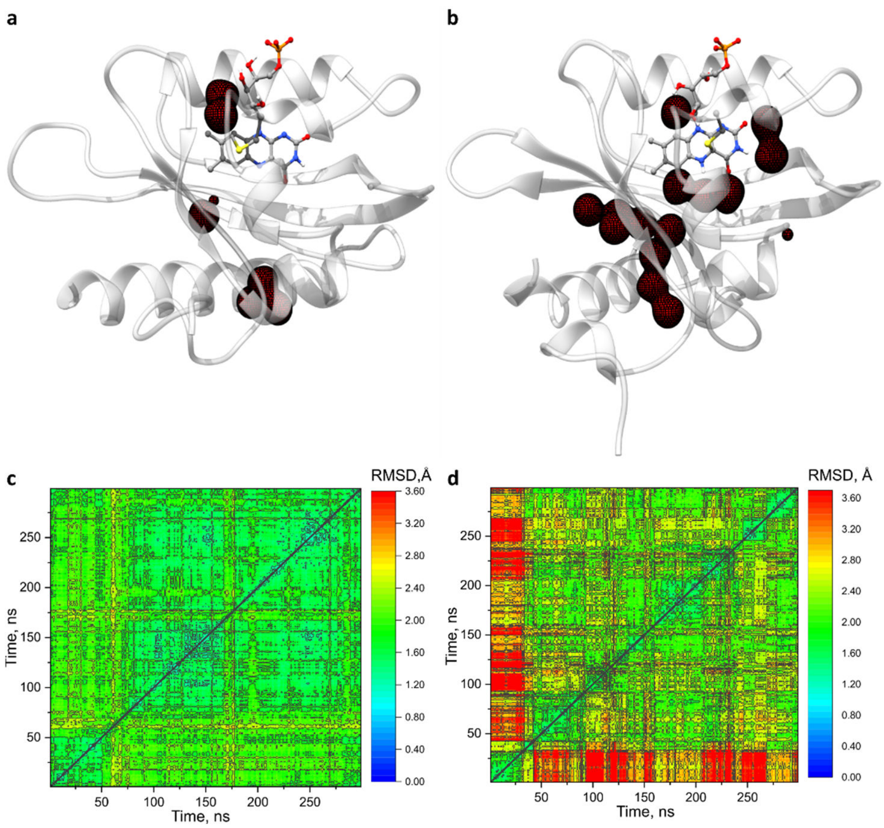

Suppl. Fig. 13 - **a-b**, Internal water density maps of *As*LOV2 wild-type derived from a 300 ns classical molecular dynamics (MD) run (see Suppl. Fig. 12). The red mesh denotes a density level of 0.3 water molecules per Å^3. Panels a and b show maps for the dark-adapted and light-adapted states of *As*LOV2 wild-type, respectively. **c-d**, Pairwise root mean square deviation between snapshots from a 300 ns MD trajectory of *As*LOV2 Q513L in the dark-adapted (panel c) and light-adapted states (panel d).

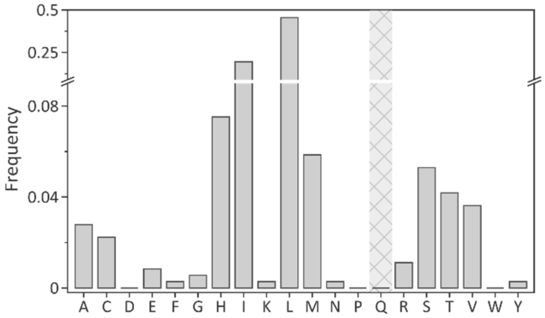

Suppl. Fig. 14 - Histogram of amino acids replacing the conserved glutamine in naturally occurring $LOV^{\Delta Q}$ receptors.

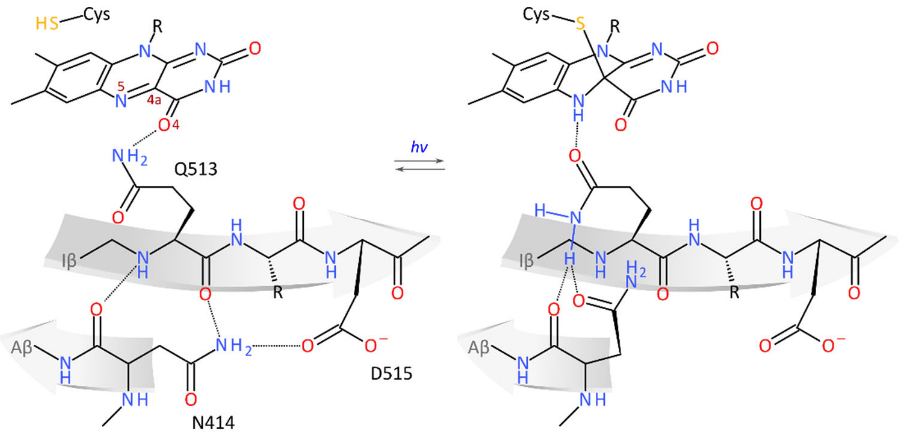

Suppl. Fig. 15 - Signal transduction in light-oxygen-voltage (LOV) receptors, exemplified for the *A. sativa* phototropin 1 LOV2 domain. The Lewis formulae show the flavin nucleotide chromophore and surrounding residues of the dark-adapted (left) and light-adapted states (right). Light-induced thioadduct formation between the flavin C4a atom and the conserved cysteine (C450) residue entails flavin protonation at the N5 atom. In response, a conserved glutamine (Q513) rotates its sidechain to satisfy hydrogen bonding, thus propagating conformational changes towards the LOV β-sheet. Hydrogen-bonding rearrangements involving N414, Q513, and D515 lead to a weakening of the β-sheet and structural elements associating with its outer face.

Supplementary Tables

Supplementary Table 1. Data collection and refinement statistics for X-ray crystallography of *As*LOV2 wild type.

	*As*LOV2 dark[a]	*As*LOV2 light[a]
**Data collection**		
Space group	P 2_1 2_1 2_1	P 2_1 2_1 2_1
Cell dimensions		
a, *b*, *c* (Å)	35.5, 56.4, 66.7	35.6, 56.0, 66.5
α, *β*, *γ* (°)	90, 90, 90	90, 90, 90
Resolution (Å)	43.07 – 1.00 (1.06 – 1.00)[b]	42.85 – 1.09 (1.16 – 1.09)
R_{meas}	0.081 (1.653)	0.075 (1.796)
I / σI	13.50 (1.22)	12.04 (1.02)
Completeness (%)	93.1 (86.5)	99.2 (98.0)
Redundancy	7.5 (7.6)	7.0 (6.2)
$CC_{1/2}$	0.999 (0.510)	0.999 (0.450)
**Refinement**		
Resolution (Å)	43.07 – 1.00 (1.03 – 1.00)	42.84 – 1.09 (1.12 – 1.09)
No. reflections	64,477 (4,481)	53,766 (4,020)
R_{work} / R_{free}	0.114 (0.320) / 0.148 (0.308)	0.123 (0.328) / 0.161 (0.387)
No. atoms		
Protein	1,186	1,186
Ligand/ion	117	89
Water	183	195
B-factors		
Protein	10.86	15.25
Ligand/ion	22.05	23.78
Water	26.26	29.75
R.m.s deviations		
Bond lengths (Å)	0.013	0.012
Bond angles (°)	1.80	1.82
PDB deposition	7pgx	7pgy

[a] Data were collected from a single crystal each.

[b] Values in parentheses are for highest-resolution shell.

Supplementary Table 2. Data collection and refinement statistics for X-ray crystallography of *As*LOV2 Q513L.

	*As*LOV2 Q513L dark[a]	*As*LOV2 Q513L light[a]
**Data collection**		
Space group	$P\ 2_1\ 2_1\ 2_1$	$P\ 2_1\ 2_1\ 2_1$
Cell dimensions		
a, *b*, *c* (Å)	35.6, 54.6, 66.5	35.3, 56.0, 66.8
α, *β*, *γ* (°)	90, 90, 90	90, 90, 90
Resolution (Å)	42.17 – 0.90 (0.95 – 0.90)[b]	42.92 – 0.98 (1.04 – 0.98)
R_{meas}	0.075 (1.908)	0.054 (2.19)
$I / \sigma I$	13.81 (0.99)	14.27 (0.74)
Completeness (%)	99.0 (97.6)	99.7 (98.3)
Redundancy	7.2 (6.9)	6.9 (6.1)
$CC_{1/2}$	1.000 (0.371)	0.999 (0.303)
**Refinement**		
Resolution (Å)	42.17 – 0.90 (0.92 – 0.90)	42.92 – 0.98 (1.01 – 0.98)
No. reflections	90,644 (6,691)	72,710 (5,330)
R_{work} / R_{free}	0.115 (0.331) / 0.139 (0.328)	0.112 (0.382) / 0.136 (0.384)
No. atoms		
Protein	1,185	1,185
Ligand/ion	119	62
Water	215	217
B-factors		
Protein	9.30	14.50
Ligand/ion	17.22	18.54
Water	22.23	30.20
R.m.s deviations		
Bond lengths (Å)	0.013	0.013
Bond angles (°)	1.99	2.04
PDB deposition	7pgz	7ph0

[a] Data were collected from a single crystal each.

[b] Values in parentheses are for highest-resolution shell.

Supplementary Table 3. Oligonucleotide primers used in this study.

Id	Sequence	Purpose
YF1_Q123X-rev	5'- AAT ACC GAC AAA ATA CGT TTT ATC CTC TAT TTC CAT T -3'	Exchange YF1 Gln123→Xaa
YF1_Q123L-fwd	5'- CTG AAT GAT ATC ACC GAG CAC CAG C -3'	Exchange YF1 Gln123→Leu
YF1_Q123A-fwd	5'- GCG AAT GAT ATC ACC GAG CAC CAG C -3'	Exchange YF1 Gln123→Ala
YF1_Q123C-fwd	5'- TGC AAT GAT ATC ACC GAG CAC CAG C -3'	Exchange YF1 Gln123→Cys
YF1_Q123D-fwd	5'- GAC AAT GAT ATC ACC GAG CAC CAG C -3'	Exchange YF1 Gln123→Asp
YF1_Q123E-fwd	5'- GAG AAT GAT ATC ACC GAG CAC CAG C -3'	Exchange YF1 Gln123→Glu
YF1_Q123F-fwd	5'- TTT AAT GAT ATC ACC GAG CAC CAG C -3'	Exchange YF1 Gln123→Phe
YF1_Q123G-fwd	5'- GGC AAT GAT ATC ACC GAG CAC CAG C -3'	Exchange YF1 Gln123→Gly
YF1_Q123H-fwd	5'- CAC AAT GAT ATC ACC GAG CAC CAG C -3'	Exchange YF1 Gln123→His
YF1_Q123I-fwd	5'- ATA AAT GAT ATC ACC GAG CAC CAG C -3'	Exchange YF1 Gln123→Ile
YF1_Q123K-fwd	5'- AAA AAT GAT ATC ACC GAG CAC CAG C -3'	Exchange YF1 Gln123→Lys
YF1_Q123M-fwd	5'- ATG AAT GAT ATC ACC GAG CAC CAG C -3'	Exchange YF1 Gln123→Met
YF1_Q123N-fwd	5'- AAC AAT GAT ATC ACC GAG CAC CAG C -3'	Exchange YF1 Gln123→Asn
YF1_Q123P-fwd	5'- CCG AAT GAT ATC ACC GAG CAC CAG C -3'	Exchange YF1 Gln123→Pro
YF1_Q123R-fwd	5'- CGC AAT GAT ATC ACC GAG CAC CAG C -3'	Exchange YF1 Gln123→Arg
YF1_Q123S-fwd	5'- AGC AAT GAT ATC ACC GAG CAC CAG C -3'	Exchange YF1 Gln123→Ser
YF1_Q123T-fwd	5'- ACG AAT GAT ATC ACC GAG CAC CAG C -3'	Exchange YF1 Gln123→Thr
YF1_Q123V-fwd	5'- GTG AAT GAT ATC ACC GAG CAC CAG C -3'	Exchange YF1 Gln123→Val
YF1_Q123W-fwd	5'- TGG AAT GAT ATC ACC GAG CAC CAG C -3'	Exchange YF1 Gln123→Trp
YF1_Q123Y-fwd	5'- TAT AAT GAT ATC ACC GAG CAC CAG C -3'	Exchange YF1 Gln123→Tyr
FixJ-19 fwd NdeI	5’-GCT CAG CAT ATG ACG ACC AAG GGA CAT ATC TAC G-3’	Subcloning *Bj*FixJ into pET19-SUMO
FixJ-19 rev BamHI	5'- CCC TAT GGG ATC CTC AAT CGT TGA GCA TGC -3'	Subcloning *Bj*FixJ into pET19-SUMO
PAL_Q347L-fwd	5‘- CAT TAC ATT GGT TAT CTG CTG GAT GTG ACC GAA C -3‘	Exchange *Nm*PAL Gln347→Leu
PAL_Q347L-rev	5'- GTT CGG TCA CAT CCA GCA GAT AAC CAA TGT AAT G -3'	Exchange *Nm*PAL Gln347→Leu
PAL_Q347H-fwd	5‘- CAT TGG TTA T CAT CT GGA TGT G -3‘	Exchange *Nm*PAL Gln347→His
PAL_Q347H-rev	5'- CAC ATC CAG ATG ATA ACC AAT G -3'	Exchange *Nm*PAL Gln347→His
PAL_Q347P-fwd	5‘- CAT TAC ATT GGT TAT CCG CTG GAT GTG ACC GAA C -3‘	Exchange *Nm*PAL Gln347→Pro
PAL_Q347P-rev	5'- GTT CGG TCA CAT CCA GCG GAT AAC CAA TGT AAT G -3'	Exchange *Nm*PAL Gln347→Pro

AsLOV2-fwd	5'- GAA GCG CAT CGC GAA CAG ATC GGT GGT GGT GAA TTT CTG GCA ACC ACA CTG -3'	Cloning of codon-optimized *As*LOV2 domain
AsLOV2-rev	5'- CCA GTG TGG TTG CCA GAA ATT CAC CAC CAC CGA TCT GTT CGC GAT GC -3'	Cloning of codon-optimized *As*LOV2 domain
AsLOV2_C450A-fwd	5'- GAA ATT CTG GGT CGT AAT GCG CGT TTT CTG CAG GG -3'	Exchange *As*LOV2 Cys450→Ala
AsLOV2_C450A-rev	5'- CCC TGC AGA AAA CGC GCA TTA CGA CCC AGA ATT TC -3'	Exchange *As*LOV2 Cys450→Ala
AsLOV2_Q513L-fwd	5'- GTA TTT TAT CGG TGT TCT GCT GGA TGG CAC C -3'	Exchange *As*LOV2 Gln513→Leu
AsLOV2_Q513L-rev	5'- GGT GCC ATC CAG CAG AAC ACC GAT AAA ATA C -3'	Exchange *As*LOV2 Gln513→Leu
AsLOV2_Q513D-fwd	5'- GTA TTT TAT CGG TGT TGA TCT GGA TGG CAC C -3'	Exchange *As*LOV2 Gln513→Asp
AsLOV2_Q513D-rev	5'- GGT GCC ATC CAG ATC AAC ACC GAT AAA ATA C -3'	Exchange *As*LOV2 Gln513→Asp
AsLOV2_Q513H-fwd	5'- GTA TTT TAT CGG TGT TCA TCT GGA TGG CAC C -3'	Exchange *As*LOV2 Gln513→His
AsLOV2_Q513H-rev	5'- GGT GCC ATC CAG ATG AAC ACC GAT AAA ATA C -3'	Exchange *As*LOV2 Gln513→His
AsLOV2_delta_Aprime	5- GCG CAT CGC GAA CAG ATC GGT GGT ATC GAA AAA AAC TTT GTT ATT ACC GAT CCG CGT C -3'	Deletion of A'α helix in *As*LOV2
AsLOV2_delta_J	5'- GGT GTT CTG CTG GAT GGC ACC TAA ATGG-GACCCGGGGTCGACCTCGAGGGATCC -3'	Deletion of Jα helix in *As*LOV2
Meso-fwd	5'-CTT TAA GAA GGA GAT ATA CAT ATG ACA GAG ATT TTC AAG GCC GGG -3'	Amplification of LOV$^{\Delta Q}$-GGDEF from *Mesorhizobium loti*
Meso-rev	5'- GTG GTG GTG CTC GAG AAC CAG CGA TTC AAA TCT TGC AGC G -3	Amplification of LOV$^{\Delta Q}$-GGDEF from *Mesorhizobium loti*
Meso-LOV-GGDEF fwd	5'- CAC CAT CAC CAT CAC CAT CAT ACG GGG ATG ACC GAA ATC TTT AAA GC -3'	Cloning of codon-optimized LOV$^{\Delta Q}$-GGDEF from *Mesorhizo-bium loti*
Meso-LOV-GGDEF rev	5'- GAG TCG CGG CCG TTA GGT CAT ACG CAG ACG ATT ACG G -3'	Cloning of codon-optimized LOV$^{\Delta Q}$-GGDEF from *Mesorhizo-bium loti*
Meso_M140Q-fwd	5'- CAG CGT GAT GTT ACC GCA AGC CG -3'	Exchange *M. loti* LOV$^{\Delta Q}$-GGDEF Met123→Gln
Meso_M140Q-rev	5'- CAG ACC AAT GTA ATG TGT CGG TTC ACC -3'	Exchange *M. loti* LOV$^{\Delta Q}$-GGDEF Met123→Gln

Supplementary References

1. Harper, S. M., Neil, L. C. & Gardner, K. H. Structural basis of a phototropin light switch. *Science* **301**, 1541–1544 (2003).
2. Weber, A. M. *et al.* A blue light receptor that mediates RNA binding and translational regulation. *Nat Chem Biol* **15**, 1085–1092 (2019).
3. Möglich, A., Ayers, R. A. & Moffat, K. Design and signaling mechanism of light-regulated histidine kinases. *J. Mol. Biol.* **385**, 1433–1444 (2009).
4. Losi, A., Quest, B. & Gärtner, W. Listening to the blue: the time-resolved thermodynamics of the bacterial blue-light receptor YtvA and its isolated LOV domain. *Photochem Photobiol Sci* **2**, 759–66 (2003).
5. Alexandre, M. T. A., Arents, J. C., van Grondelle, R., Hellingwerf, K. J. & Kennis, J. T. M. A Base-Catalyzed Mechanism for Dark State Recovery in the Avena sativa Phototropin-1 LOV2 Domain. *Biochemistry* **46**, 3129–3137 (2007).
6. Ohlendorf, R., Vidavski, R. R., Eldar, A., Moffat, K. & Möglich, A. From dusk till dawn: one-plasmid systems for light-regulated gene expression. *J. Mol. Biol.* **416**, 534–542 (2012).
7. Hennemann, J. *et al.* Optogenetic Control by Pulsed Illumination. *Chembiochem* **19**, 1296–1304 (2018).
8. Halavaty, A. S. & Moffat, K. N- and C-terminal flanking regions modulate light-induced signal transduction in the LOV2 domain of the blue light sensor phototropin 1 from Avena sativa. *Biochemistry* **46**, 14001–14009 (2007).
