# Supplementary material for: Signal transduction in light-oxygen-voltage receptors lacking the active-site glutamine
Source: Nat Commun. 2022 May 12;13:2618. doi: 10.1038/s41467-022-30252-4 (PMC9098866; doi:10.1038/s41467-022-30252-4)

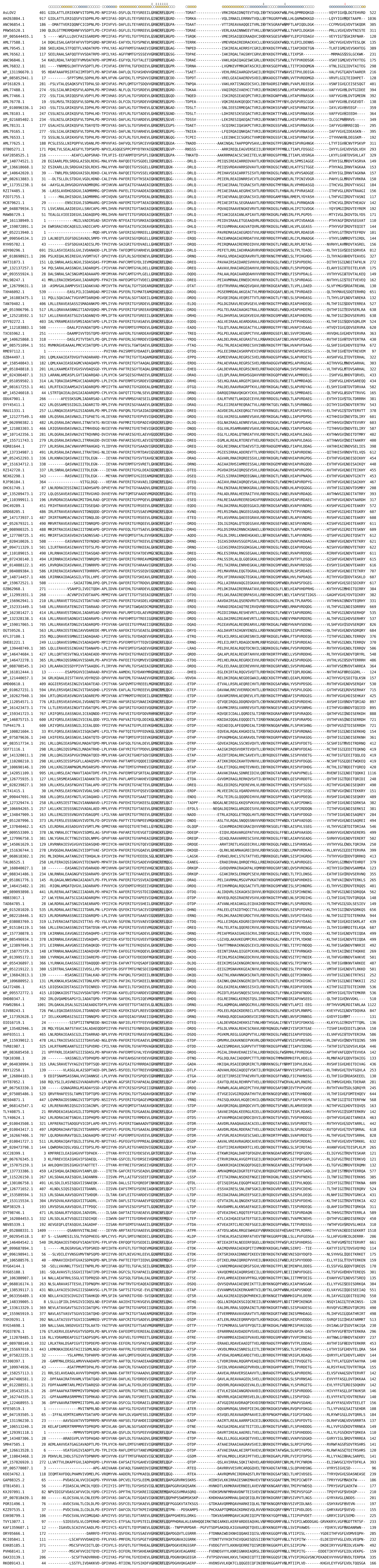

Supplement: Supplementary file 5 — Supplementary Data 3 [file 41467_2022_30252_MOESM5_ESM.pdf]
