# Supplementary material for: Signal transduction in light-oxygen-voltage receptors lacking the active-site glutamine
Source: Nat Commun. 2022 May 12;13:2618. doi: 10.1038/s41467-022-30252-4 (PMC9098866; doi:10.1038/s41467-022-30252-4)
Supplement: Supplementary file 6 — Reporting Summary [file 41467_2022_30252_MOESM6_ESM.pdf]

nature portfolio

Corresponding author(s): Möglich, Andreas

Last updated by author(s): Mar 11, 2022

Reporting Summary

Nature Portfolio wishes to improve the reproducibility of the work that we publish. This form provides structure for consistency and transparency in reporting. For further information on Nature Portfolio policies, see our Editorial Policies and the Editorial Policy Checklist.

Statistics

For all statistical analyses, confirm that the following items are present in the figure legend, table legend, main text, or Methods section.

n/a	Confirmed	
☐	☒	The exact sample size (*n*) for each experimental group/condition, given as a discrete number and unit of measurement
☐	☒	A statement on whether measurements were taken from distinct samples or whether the same sample was measured repeatedly
☒	☐	The statistical test(s) used AND whether they are one- or two-sided<br>*Only common tests should be described solely by name; describe more complex techniques in the Methods section.*
☒	☐	A description of all covariates tested
☒	☐	A description of any assumptions or corrections, such as tests of normality and adjustment for multiple comparisons
☐	☒	A full description of the statistical parameters including central tendency (e.g. means) or other basic estimates (e.g. regression coefficient) AND variation (e.g. standard deviation) or associated estimates of uncertainty (e.g. confidence intervals)
☒	☐	For null hypothesis testing, the test statistic (e.g. *F*, *t*, *r*) with confidence intervals, effect sizes, degrees of freedom and *P* value noted<br>*Give P values as exact values whenever suitable.*
☒	☐	For Bayesian analysis, information on the choice of priors and Markov chain Monte Carlo settings
☒	☐	For hierarchical and complex designs, identification of the appropriate level for tests and full reporting of outcomes
☒	☐	Estimates of effect sizes (e.g. Cohen's *d*, Pearson's *r*), indicating how they were calculated

Our web collection on statistics for biologists contains articles on many of the points above.

Software and code

Policy information about availability of computer code

Data collection	XDS build 20200131, XDSapp 2.0
Data analysis	pointless 1.11.17, phaser 2.5.6, Coot 0.8.9.2, Refmac 5.8.0267, lsqkab 7.1.013, scaleit 7.1.013, PyMOL 2.1.0, ClustalX 2.1, WebLogo 3.7, fit-o-mat 0.873, AMBER 18, UCSF Chimera 1.15, VMD 1.9.2, APBS 3.0, VolMap 1.1

For manuscripts utilizing custom algorithms or software that are central to the research but not yet described in published literature, software must be made available to editors and reviewers. We strongly encourage code deposition in a community repository (e.g. GitHub). See the Nature Portfolio guidelines for submitting code & software for further information.

Data

Policy information about availability of data

All manuscripts must include a data availability statement. This statement should provide the following information, where applicable:

- Accession codes, unique identifiers, or web links for publicly available datasets
- A description of any restrictions on data availability
- For clinical datasets or third party data, please ensure that the statement adheres to our policy

Atomic coordinates and structure-factor amplitudes have been deposited in the Protein Data Bank under accession codes 7pgx [http://doi.org/10.2210/pdb7pgx/pdb] (AsLOV2 wild-type, dark), 7pgy [http://doi.org/10.2210/pdb7pgy/pdb] (wild-type, light), 7pgz [http://doi.org/10.2210/pdb7pgz/pdb] (Q513L, dark), and 7ph0 [http://doi.org/10.2210/pdb7ph0/pdb] (Q513L, light).

MTZ files underpinning the light-dark difference electron density maps (see Suppl. Fig. S8) are provided as Supplementary Data.

Other data generated in this study have been deposited in the Zenodo database under accession code 6341885 [https://doi.org/10.5281/zenodo.6341885].

A Python script for sequence analysis of glutamine-deficient LOV receptors is available from https://github.com/TheAngulion/LOVfilterQ.

Field-specific reporting

Please select the one below that is the best fit for your research. If you are not sure, read the appropriate sections before making your selection.

☒ Life sciences ☐ Behavioural & social sciences ☐ Ecological, evolutionary & environmental sciences

For a reference copy of the document with all sections, see nature.com/documents/nr-reporting-summary-flat.pdf

Life sciences study design

All studies must disclose on these points even when the disclosure is negative.

Sample size	For all measurements, at least three biological replicates (i.e. independent samples) were analyzed and averaged. No statistical tests were used to determine sample size. The sample sizes are stated for each experiment. They were determined based on the size and distribution of the measurable differences in the experimental observable.
Data exclusions	No data were excluded.
Replication	All experiments were repeated at least twice with similar outcome. Details are provided in the figure legends.
Randomization	Randomization was not applied as study did not involve group comparison.
Blinding	Blinding was not applied as study did not involve group comparison.

Reporting for specific materials, systems and methods

We require information from authors about some types of materials, experimental systems and methods used in many studies. Here, indicate whether each material, system or method listed is relevant to your study. If you are not sure if a list item applies to your research, read the appropriate section before selecting a response.

Materials & experimental systems

n/a	Involved in the study	
☒	☐	Antibodies
☐	☒	Eukaryotic cell lines
☒	☐	Palaeontology and archaeology
☒	☐	Animals and other organisms
☒	☐	Human research participants
☒	☐	Clinical data
☒	☐	Dual use research of concern

Methods

n/a	Involved in the study	
☒	☐	ChIP-seq
☒	☐	Flow cytometry
☒	☐	MRI-based neuroimaging

Eukaryotic cell lines

Policy information about cell lines

Cell line source(s)	HeLa cells, purchased from CLS Cell Lines Service GmbH, Eppelheim, Germany
Authentication	The identity of the HeLa cells was verified by the vendor via STR DNA profiling.
Mycoplasma contamination	The absence of Mycoplasma contamination was confirmed by the vendor.
Commonly misidentified lines (See ICLAC register)	No such lines were used in this study.
